# Evaluation of Traditional Prognostic Factors for Stage I-III Colorectal Cancer Patients Who Survived for Over Five Years After Surgery

**DOI:** 10.3389/fonc.2021.618820

**Published:** 2021-09-09

**Authors:** Dakui Luo, Yufei Yang, Zezhi Shan, Qi Liu, Sanjun Cai, Qingguo Li, Xinxiang Li

**Affiliations:** ^1^Department of Colorectal Surgery, Fudan University Shanghai Cancer Center, Shanghai, China; ^2^Department of Oncology, Shanghai Medical College, Fudan University, Shanghai, China

**Keywords:** colorectal cancer, survive over five years, prognostic factors, FUSCC, COX

## Abstract

The aim of this study was to explore the prognostic factors in stage I-III colorectal cancer (CRC) patients who had survived for over five years. A total of 9754 stage I-III CRC patients who received curative surgery in the Department of Colorectal Surgery, Fudan University Shanghai Cancer Center were enrolled in this study. Of them, 3640 patients had survived for over five years after surgery. Univariate and multivariate Cox regression analyses were performed in the entire cohort and those who had survived for over five years. Compared with patients in the entire cohort, patients who had survived for over five years were more likely to be younger, have less disease of signet ring cell histology, perineural invasion and vascular invasion, more well differentiated tumors and stage I disease. In the entire cohort, increased age, signet ring cell, poor differentiation, more advanced pathological stage, perineural invasion and vascular invasion were inversely associated with disease-free survival (DFS) and overall survival (OS) using multivariable Cox regression analyses. Only age, pathological stage and perineural invasion remained significant in patients who had survived for over five years. Moreover, tumor location was an independent factor for OS in this subgroup. Predictors for prognosis of CRC change over time. Age, pathological stage and perineural invasion deserve more attention among patients who have survived for over five years.

## Introduction

Colorectal cancer (CRC) is one of the most common malignancies and cause of cancer-related deaths worldwide (1). Several factors have shown prognostic and predictive values to facilitate decisions regarding adjuvant therapy and post-treatment surveillance. Of them, the tumor, lymph node, metastasis (TNM) staging system has been used as the fundamental classification for predicting the prognosis of CRC patients (2). Previous studies have shown that 80% of recurrences occurred in the first three years and 95% of recurrences occurred in the first five years after surgical resection (3). Recurrence and cancer-related mortality are rarely found in patients who have survived over five years (4). The risk of relapse and cancer-specific death changes over time. Similarly, the role of prognostic factors may change for patients who have survived for a long time.

To the best of our knowledge, whether conventional prognostic factors could be used to effectively evaluate survival of patients who have survived for a long period of time after surgery has not been investigated. The purpose of this study was to determine if the prognostic factors changed over a time frame of five years.

## Materials and Methods

### Study Population

A total of 13765 CRC patients were identified from the Fudan University Shanghai Cancer Center (FUSCC) database between January 2008 between May 2018. The inclusion and exclusion criteria were as follows: (1) Patients underwent curative surgery; (2) Patients were diagnosed with stage I-III primary CRC and patients with stage Tis, stage IV or undetermined TNM stage were excluded; (3) Patients who underwent neoadjuvant therapy were excluded. (4) There is no age limit. Finally, 9754 patients were enrolled in this study. Of them, 3640 patients had survived for over five years after surgery. The following variables were extracted from the FUSCC database: age at diagnosis; gender; tumor location; histologic type; differentiation; American Joint Committee On Cancer (AJCC) stage; perineural invasion; vascular invasion; survival data. This study was approved by the Ethic Committee and Institutional Review Board of the FUSCC, and written informed consent was obtained from all the patients.

### Treatment and Follow-Up

All patients received standard radical surgery based on the principles of oncology. Adjuvant chemotherapy was administrated to “high risk” stage II and all stage III patients who could tolerate the treatment. Generally, stage II patients without high risk factors can be treated with capecitabine alone or placed under observation while patients with high-risk stage II disease and stage III disease can be considered for adjuvant chemotherapy with CapeOX (oxaliplatin and capecitabine). Adjuvant radiotherapy was adopted in selected rectal cancer patients. Medical records review, telephonic follow-ups and death registry data linkage were employed for collecting survival data. The last follow-up date was November 30, 2019.

### Statistical Analysis

Categorical variables were analyzed by the chi-squared test. Univariate and multivariate Cox regression analyses were performed to identify the prognostic factors associated with DFS and OS. All statistical analyses were performed with SPSS 25.0. Kaplan-Meier curves were performed using R (version 3.6.3).

## Results

### Characteristics of Patients Who Had Survived for Over Five Years

A total of 9754 stage I-III CRC patients who underwent curative surgery without receiving neoadjuvant therapy were enrolled in this study. The overall recurrence rate after curative surgery was 24.0% (2341/9754). Of them, 3640 patients had survived for over five years after surgery. 274 patients experienced recurrence over five years after surgery. Compared with patients in the entire cohort, patients who had survived over five years were more likely to be younger, had less disease of signet ring cell histology, perineural invasion and vascular invasion, more well differentiated tumors and stage I disease (**Table 1**).

**Table 1:** Characteristics of stage I-III CRC patients who received radical surgery in FUSCC between 2008 and 2018 (N = 9754) and patients who survived 5 years following surgery (n = 3640).

Characteristics	Entire cohort	5-year survivors	P value
**Age**	59.62 ± 12.0	58.73 ± 11.7	0.022
**Gender**			0.764
Male	5741 (58.9)	2132 (58.6)	
Female	4013 (41.1)	1508 (41.4)	
**Location**			0.455
Rectum	4975 (51.0)	1883 (51.7)	
Colon	4779 (49.0)	1757 (48.3)	
**Histologic type**			<0.001
Adenocarcinoma	8453 (86.7)	3152 (86.6)	
Mucinous	1129 (11.6)	456 (12.5)	
Signet ring cell	172 (1.7)	32 (0.9)	
**Differentiation**			<0.001
Well	172 (1.8)	77 (2.1)	
Moderate	7196 (73.8)	2825 (77.6)	
Poor	2170 (22.2)	653 (17.9)	
Unknown	216 (2.2)	85 (2.3)	
**TNM stage**			<0.001
I	1922 (19.7)	861 (23.7)	
II	3505 (35.9)	1343 (36.9)	
III	4327 (44.4)	1436 (39.5)	
**Perineural invasion**			<0.001
Negative	7680 (78.7)	3103 (85.2)	
Positive	2074 (21.3)	537 (14.8)	
**Vascular invasion**			<0.001
Negative	7443 (76.3)	2983 (82.0)	
Positive	2311 (23.7)	657 (18.0)	

### Prognosis Analysis of the Entire Cohort

In the entire cohort, univariate analyses suggested that age at diagnosis, histologic type, differentiation, TNM stage, perineural invasion and vascular invasion were associated with DFS and OS. Moreover, gender was also associated with DFS. Increased age, signet ring cell, poor differentiation, more advanced pathological stage, perineural invasion and vascular invasion were inversely associated with (DFS) and OS using multivariable Cox regression analyses. Gender was an independent prognostic factor for DFS (**Tables 2** and **3**).

**Table 2:** Univariable and multivariable Cox regression of disease-free survival in entire cohort.

Characteristics	Univariable Cox Regression		Multivariable Cox Regression	
	HR (95% CI)	P	HR (95% CI)	P
**Age**	1.015 (1.011-1.018)	<0.001	1.019 (1.015-1.022)	<0.001
**Gender**				
Male	Reference		Reference	
Female	0.895 (0.825-0.971)	0.008	0.902 (0.832-0.979)	0.013
**Location**				
Rectum	Reference		Reference	
Colon	1.077 (0.995-1.166)	0.067	1.043 (0.962-1.131)	0.305
**Histologic type**				
Adenocarcinoma	Reference		Reference	
Mucinous	1.117 (0.993-1.258)	0.066	0.995 (0.872-1.134)	0.935
Signet ring cell	2.449 (1.953-3.071)	<0.001	1.410 (1.106-1.798)	0.006
**Differentiation**				
Poor	Reference		Reference	
Moderate	0.611 (0.559-0.668)	<0.001	0.783 (0.709-0.865)	<0.001
Well	0.387 (0.263-0.568)	<0.001	0.671 (0.454-0.991)	0.045
Unknown	/		/	
**TNM stage**				
I	Reference		Reference	
II	1.373 (1.193-1.579)	<0.001	1.213 (1.051-1.400)	0.008
III	2.844 (2.503-3.232)	<0.001	2.057 (1.792-2.362)	<0.001
**Perineural invasion**				
Negative	Reference		Reference	
Positive	2.174 (1.996-2.368)	<0.001	1.611 (1.470-1.766)	<0.001
**Vascular invasion**				
Negative	Reference		Reference	
Positive	2.153 (1.982-2.339)	<0.001	1.390 (1.265-1.528)	<0.001

**Table 3:** Univariable and multivariable Cox regression of overall survival in entire cohort.

Characteristics	Univariable Cox Regression		Multivariable Cox Regression	
	HR (95% CI)	P	HR (95% CI)	P
**Age**	1.030 (1.025-1.034)	<0.001	1.036 (1.032-1.041)	<0.001
**Gender**				
Male	Reference		Reference	
Female	0.909 (0.824-1.003)	0.058	0.933 (0.845-1.030)	0.167
**Location**				
Rectum	Reference		Reference	
Colon	1.055 (0.959-1.162)	0.272	0.989 (0.897-1.091)	0.824
**Histologic type**				
Adenocarcinoma	Reference		Reference	
Mucinous	1.209 (1.051-1.390)	0.008	1.060 (0.908-1.237)	0.458
Signet ring cell	3.516 (2.745-4.503)	<0.001	2.025 (1.549-2.647)	<0.001
**Differentiation**				
Poor	Reference		Reference	
Moderate	0.532 (0.479-0.591)	<0.001	0.711 (0.632-0.800)	<0.001
Well	0.363 (0.230-0.574)	<0.001	0.685 (0.430-1.090)	0.111
Unknown	/		/	
**TNM stage**				
I	Reference		Reference	
II	1.423 (1.185-1.709)	<0.001	1.252 (1.039-1.509)	0.018
III	3.541 (3.002-4.176)	<0.001	2.528 (2.119-3.016)	<0.001
**Perineural invasion**				
Negative	Reference		Reference	
Positive	2.293 (2.069-2.543)	<0.001	1.621 (1.452-1.809)	<0.001
**Vascular invasion**				
Negative	Reference		Reference	
Positive	2.512 (2.276-2.772)	<0.001	1.501 (1.342-1.678)	<0.001

### Prognosis Analysis of Patients Who Had Survived for Over Five Years

**Figure 1:**
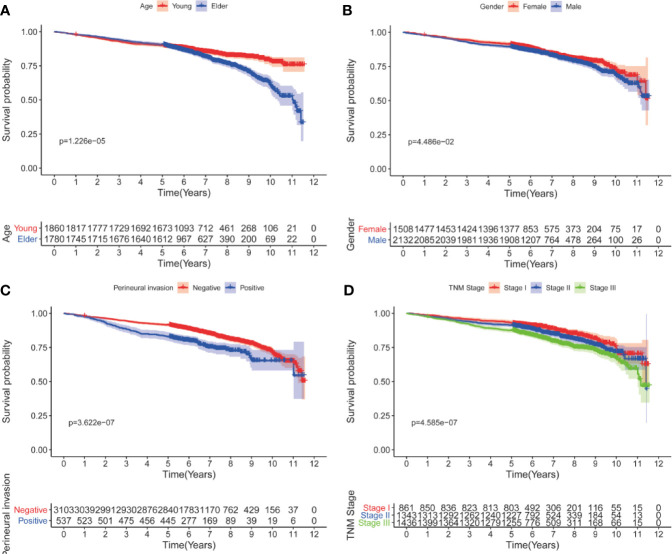
Kaplan-Meier curves for DFS after five years according to clinicopathological factors. **(A)** age; **(B)** gender; **(C)** perineural invasion; **(D)** pathological stage.

**Figure 2:**
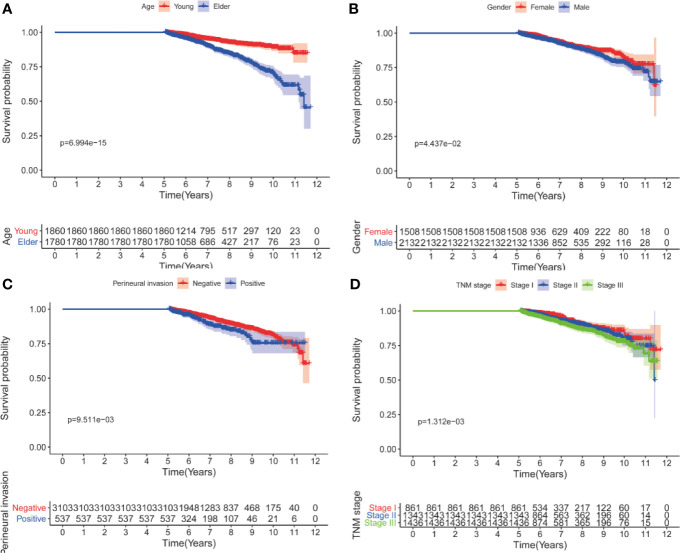
Kaplan-Meier curves for OS after five years according to clinicopathological factors. **(A)** age; **(B)** gender; **(C)** perineural invasion; **(D)** pathological stage.

Univariate analyses showed that only age at diagnosis, gender, pathological stage and perineural invasion remained significant in patients who had survived for over five years (**Tables 4** and **5**). Kaplan-Meier curves were plotted based on the above prognostic factors (**Figures 1** and **2**). Multivariable Cox regression analyses showed that age, pathological stage and perineural invasion were independent prognostic factors for predicting DFS and OS. Moreover, tumor location was an independent factor for predicting OS in this subgroup.

**Table 4:** Univariable and multivariable Cox regression of disease-free survival in patients who survived over 5 years after surgery.

Characteristics	Univariable Cox Regression		Multivariable Cox Regression	
	HR (95% CI)	P	HR (95% CI)	P
**Age**	1.017 (1.010-1.024)	<0.001	1.019 (1.012-1.026)	<0.001
**Gender**				
Male	Reference		Reference	
Female	0.849 (0.723-0.996)	0.045	0.872 (0.742-1.025)	0.096
**Location**				
Rectum	Reference		Reference	
Colon	0.992 (0.849-1.160)	0.921	0.952 (0.811-1.117)	0.548
**Histologic type**				
Adenocarcinoma	Reference		Reference	
Mucinous	1.088 (0.870-1.360)	0.459	1.060 (0.826-1.361)	0.648
Signet ring cell	0.589 (0.189-1.834)	0.361	0.486 (0.153-1.542)	0.221
**Differentiation**				
Poor	Reference		Reference	
Moderate	0.933 (0.763-1.142)	0.503	0.974 (0.783-1.210)	0.810
Well	0.560 (0.284-1.103)	0.093	0.678 (0.341-1.351)	0.269
Unknown	/		/	
**TNM stage**				
I	Reference		Reference	
II	1.260 (1.004-1.581)	0.046	1.168 (0.924-1.476)	0.193
III	1.679 (1.352-2.084)	<0.001	1.588 (1.258-2.005)	<0.001
**Perineural invasion**				
Negative	Reference		Reference	
Positive	1.647 (1.356-2.001)	<0.001	1.560 (1.271-1.913)	<0.001
**Vascular invasion**				
Negative	Reference		Reference	
Positive	1.132 (0.931-1.376)	0.214	0.884 (0.714-1.095)	0.260

**Table 5:** Univariable and multivariable Cox regression of overall survival in patients who survived over 5 years after surgery.

Characteristics	Univariable Cox Regression		Multivariable Cox Regression	
	HR (95% CI)	P	HR (95% CI)	P
**Age**	1.053 (1.041-1.064)	<0.001	1.056 (1.045-1.068)	<0.001
**Gender**				
Male	Reference		Reference	
Female	0.783 (0.616-0.994)	0.045	0.829 (0.651-1.055)	0.127
**Location**				
Rectum	Reference		Reference	
Colon	0.878 (0.696-1.106)	0.269	0.776 (0.611-0.984)	0.036
**Histologic type**				
Adenocarcinoma	Reference		Reference	
Mucinous	1.075 (0.779-1.482)	0.660	1.143 (0.799-1.636)	0.464
Signet ring cell	1.128 (0.280-4.540)	0.865	1.097 (0.263-4.568)	0.899
**Differentiation**				
Poor	Reference		Reference	
Moderate	0.954 (0.703-1.294)	0.761	1.014 (0.732-1.403)	0.935
Well	0.953 (0.432-2.101)	0.905	1.150 (0.513-2.578)	0.735
Unknown	/		/	
**TNM stage**				
I	Reference		Reference	
II	1.234 (0.883-1.727)	0.219	1.203 (0.852-1.700)	0.294
III	1.624 (1.180-2.235)	0.003	1.694 (1.200-2.391)	0.003
**Perineural invasion**				
Negative	Reference		Reference	
Positive	1.480 (1.098-1.995)	0.010	1.490 (1.089-2.039)	0.013
**Vascular invasion**				
Negative	Reference		Reference	
Positive	1.144 (0.861-1.520)	0.353	0.901 (0.660-1.229)	0.511

## Discussion

CRC is characterized by heterogeneity with distinctive survival outcomes (5). Prognostic prediction of CRC largely depends on clinicopathological characteristics (6–10). TNM staging system is the fundamental prognostic factor of CRC patients. For patients with CRC, nearly 80% of recurrences emerged in the first three years. The risk of relapse and cancer-specific mortality decreased dramatically in patients who had survived for over five years. The traditional prognostic factors were identified based on 5-year survival rates postoperatively. Therefore, the prognostic factors of patients who had survived for a long period of time may change. Whether the conventional prognostic factors still precisely predict the prognosis of CRC has not been established in patients who have survived for over five years after surgery. Several previous studies have indicated that the prognostic factors for cancer-specific death change over time in different malignancies (11, 12). It is necessary to explore the long-term prognostic factors of CRC patients who had received curative surgery.

This study demonstrated that the clinical characteristics of CRC patients who had survived for over five years differed from those in entire cohort. We observed that increased age, male gender, more advanced pathological stage and perineural invasion were associated with adverse prognosis in patients who had survived for over five years following radical surgery, which was different from prognostic factors of the entire cohort. Only age, pathological stage and perineural invasion were independent prognostic factors using multivariable Cox regression analyses. The prognostic values of histologic type, differentiation and vascular invasion weakened over time and disappeared after a long period of time.

Age has been a stable predictor for stratifying risk of relapse and mortality over time. Young patients have better survival outcomes as compared to elderly patients (13). During the follow-up, young patients tend to tolerate more intensive therapy and show excellent compliance. Since the first edition of the AJCC Cancer Staging Manual was released in 1977, the pathological stage remains the most important factors for predicting prognosis of CRC patients (14), which was also observed in our study. Its predictive performance for DFS or OS remains excellent over time. The results of our study reconfirmed the importance of AJCC stage in prognostic prediction. The proportion of more advanced stage was lower in patients who had survived for over five years. Vascular and perineural invasions are important markers of tumor aggressiveness and predict poor prognosis in CRC, which were also demonstrated in our study. As expected, patients with more than five years of follow-up demonstrated less positive vascular and perineural invasions compared to the entire cohort. Intriguingly, the prognostic value of vascular invasion was missing while perineural invasion showed a robust association with poor survival. The difference between vascular invasion and perineural invasion should be consider over time during the follow-up.

This study had some inevitable limitations. First, several important prognostic factors, such as tumor size, pretreatment serum CEA level, microsatellite instability and the detailed information of adjuvant chemotherapy (15–17), were not recorded in our dataset. Second, the prognostic value of clinicopathological features change over time, we speculated that the variation occurred in patients who survive for a specific time after surgery. However, it is difficult to identify the optimal cut-off value. Hence, the long-term survival was depicted by five years in this study. Third, patients who received neoadjuvant therapy were excluded, which led to a decrease in the proportion of patients with more advanced disease, thereby resulting in selection bias. Despite the above limitations, to the best of our knowledge, this was the first large sample size study report that many conventional prognostic factors could not accurately predict DFS and OS of stage I-III CRC patients who had survived for over five years after surgery. These results will contribute to optimize the postoperative follow-up regime for patients who have survived for more than five years.

In summary, prognostic factors of CRC change over time. For patients who have survived for over five years, age, pathological stage and perineural invasion remain important prognostic factors while the others do not.

## Data Availability Statement

The raw data supporting the conclusions of this article will be made available by the authors, without undue reservation.

## Ethics Statement

The studies involving human participants were reviewed and approved by Ethical Committee and Institutional Review Board of the Fudan University Shanghai Cancer Center. The patients/participants provided their written informed consent to participate in this study.

## Author Contributions

XL and QLi conceived this study. DL, YY and QLiu improved the study design and contributed to the interpretation of results. YY and ZS collected the data. SC conducted data processing and statistical analysis. DL and ZS wrote the manuscript. QLi revised the manuscript. All authors contributed to the article and approved the submitted version.

## Funding

This work was supported by the National Natural Science Foundation of China (Grant NO. 81972260; NO. 81772599). The funders had no role in the study design, data collection and analysis, decision to publish, or preparation of the manuscript.

## Conflict of Interest

The authors declare that the research was conducted in the absence of any commercial or financial relationships that could be construed as a potential conflict of interest.

## Publisher’s Note

All claims expressed in this article are solely those of the authors and do not necessarily represent those of their affiliated organizations, or those of the publisher, the editors and the reviewers. Any product that may be evaluated in this article, or claim that may be made by its manufacturer, is not guaranteed or endorsed by the publisher.
